# Causal associations between pediatric asthma and united airways disease: a two-sample Mendelian randomization analysis

**DOI:** 10.3389/fmed.2024.1369695

**Published:** 2024-06-10

**Authors:** Tongxun Gao, Qiuhan Cai, Siyuan Hu, Rongxin Zhu, Jixuan Wang

**Affiliations:** ^1^Department of Clinical Trial Center, First Teaching Hospital of Tianjin University of Traditional Chinese Medicine, Tianjin, China; ^2^National Clinical Research Center for Chinese Medicine Acupuncture and Moxibustion, Tianjin, China

**Keywords:** Mendelian randomization, pediatric asthma, united airways disease, chronic respiratory diseases, chronic rhinitis, chronic obstructive pulmonary disease

## Abstract

**Background:**

Prior observational research has indicated a potential link between pediatric asthma and united airways disease (UAD). However, these findings could be subject to confounding factors and reverse causation. Therefore, our study utilizes Mendelian randomization (MR) method to further investigate the causal relationship between pediatric asthma and UAD.

**Methods:**

We conducted a comprehensive two-sample Mendelian randomization (MR) analysis to investigate the association between pediatric asthma and seven groups of UAD, including chronic sinusitis, chronic rhinitis, nasopharyngitis and pharyngitis, chronic diseases of tonsils and adenoids, chronic laryngitis and laryngotracheitis, chronic bronchitis, bronchiectasis, chronic obstructive pulmonary disease (COPD). The present study employed a range of methods for two-sample MR analysis, including inverse variance weighted (IVW), MR-Egger regression, Simple mode, weighted median, and weighted models. The conclusion of the MR analysis primarily relies on the IVW results, while other analytical methods are utilized as supplementary evidence to ensure result robustness in this MR analysis. And sensitivity analyses were conducted, including heterogeneity test, horizontal pleiotropy test, MR-PRESSO test, and leave-one-out analysis to validate the results.

**Results:**

The results of the MR analysis indicate significant causal effects of pediatric asthma on chronic rhinitis, nasopharyngitis and pharyngitis (IVW: OR = 1.15, 95%CI: 1.05–1.26, *p*-value = 0.003), chronic diseases of tonsils and adenoids (IVW: OR = 1.07, 95%CI: 1.00–1.15, *p*-value = 0.038), chronic bronchitis (IVW: OR = 1.51, 95%CI: 1.42–1.62, *p*-value <0.001), bronchiectasis (IVW: OR = 1.51, 95%CI: (1.30–1.75), *p*-value <0.001), and COPD (IVW: OR = 1.43, 95%CI: 1.34–1.51, *p*-value <0.001). However, no significant causal association was observed between pediatric asthma and chronic sinusitis (IVW: OR = 1.00, 95%CI: 1.00–1.00, *p*-value = 0.085), chronic laryngitis and laryngotracheitis (IVW: OR = 1.05, 95%CI: 0.90–1.21, *p*-value = 0.558).

**Conclusion:**

Our findings support a potential causal relationship between pediatric asthma and UAD, suggesting that pediatric asthma may be a potential risk factor for various UAD.

## 1 Introduction

Asthma, a prevalent chronic condition in childhood that typically manifests early in life, is a serious global health issue affecting approximately 20% of children worldwide, causing significant distress to individual patients, and imposing a substantial burden on both the community and healthcare system (1, 2). Over half of children will experience a wheezing episode by the age of 6 years (3). Fortunately, this condition demonstrates a favorable treatment response, and a substantial proportion of pediatric asthma cases resolve prior to school-age and adolescence (4). However, the clinical remission of symptoms does not necessarily equate to the complete disappearance of the underlying pathological phenomenon. In fact, despite the absence of obvious symptoms, airway hyperresponsiveness and airway inflammation may still exist, and airway remodeling can also be observed in preschool children and school-age children with asthma, and these phenomena may even have a lifelong impact on airway health (4–6).

The concept of united airways disease (UAD) posits that the airway constitutes a comprehensive anatomical and functional entity spanning from the nasal cavity to the bronchus, thereby challenging the conventional division of the respiratory tract into upper and lower regions solely based on vocal cord demarcation (7, 8). The concept of UAD was initially conceived based on the observed coexistence of allergic rhinitis and chronic sinusitis with asthma (9). However, through further research, its scope has expanded beyond these specific diseases to gradually encompass other chronic inflammatory conditions affecting the upper and/or lower respiratory tract, such as adenoid hypertrophy, bronchiectasis, and COPD (7, 10).

The risk factors for pediatric asthma and UAD overlap significantly, encompassing exposure to secondhand smoke, environmental particulate matter, household air pollution, high body mass index, infections, and immune dysfunction (1, 11). For instance, prenatal and postnatal exposure to air pollution and maternal smoking increase the risk of asthma in children (12). Recurrent respiratory infections, smoking, childhood and adolescent asthma, and early-life exposure to air pollution are potential risk factors associated with the development of chronic bronchitis in young individuals (13). Globally, smoking is the most common risk factor for COPD, followed by environmental particulate matter pollution (11). UAD is prevalent in clinical practice, supported by extensive epidemiological evidence. Asthma and sinusitis frequently coexist, particularly among children; 34–50% of sinusitis patients have comorbid asthma, and the prevalence of sinusitis in individuals with asthma can reach up to 84% (14). Allergic asthma-rhinitis phenotype typically manifests during childhood, with 40% of patients diagnosed with allergic rhinitis also presenting symptoms of asthma, and nearly all individuals with asthma exhibiting manifestations of allergic rhinitis (15). A longitudinal cohort study on asthma phenotypes from childhood to middle age demonstrates that early-onset remitting, early-onset adult remitting, early-onset persistent, and late-onset persistent phenotypes are significantly associated with the incidence of COPD by age 53 (16).

Mendelian randomization (MR) is a genetic epidemiological approach that utilizes genetic variants associated with varying exposures to evaluate their causal relationship with outcomes, aiming to mitigate confounding and potential bias arising from reverse causation (17). Genetic variants are randomly distributed during meiosis and remain unaffected by later-life diseases. Consequently, the MR method can effectively mitigate confounding factors and eliminate interference from reverse causality, making it superior to traditional observational studies (18, 19). Given the aforementioned characteristics, MR methods have gained widespread utilization in investigating the causal relationship between traits and diseases, as well as among different diseases. UAD encompasses a diverse range of diseases across various disciplines; however, clinicians often concentrate on diagnosing and treating diseases within their own specialty, overlooking the clinical manifestations and diagnosis of these diseases in other areas. This oversight hampers the improvement of diagnostic accuracy and treatment efficacy (20). We conducted this MR study to elucidate the causal relationship between pediatric asthma and UAD, encompassing chronic sinusitis, chronic rhinitis, nasopharyngitis and pharyngitis, chronic diseases of tonsils and adenoids, chronic laryngitis and laryngotracheitis, chronic bronchitis, bronchiectasis, COPD, through genetic analysis. This study provides novel insights for managing comorbidities associated with pediatric asthma as well as facilitating the diagnosis, prevention, and treatment of UAD.

## 2 Materials and methods

### 2.1 Study design

**Figure 1:**
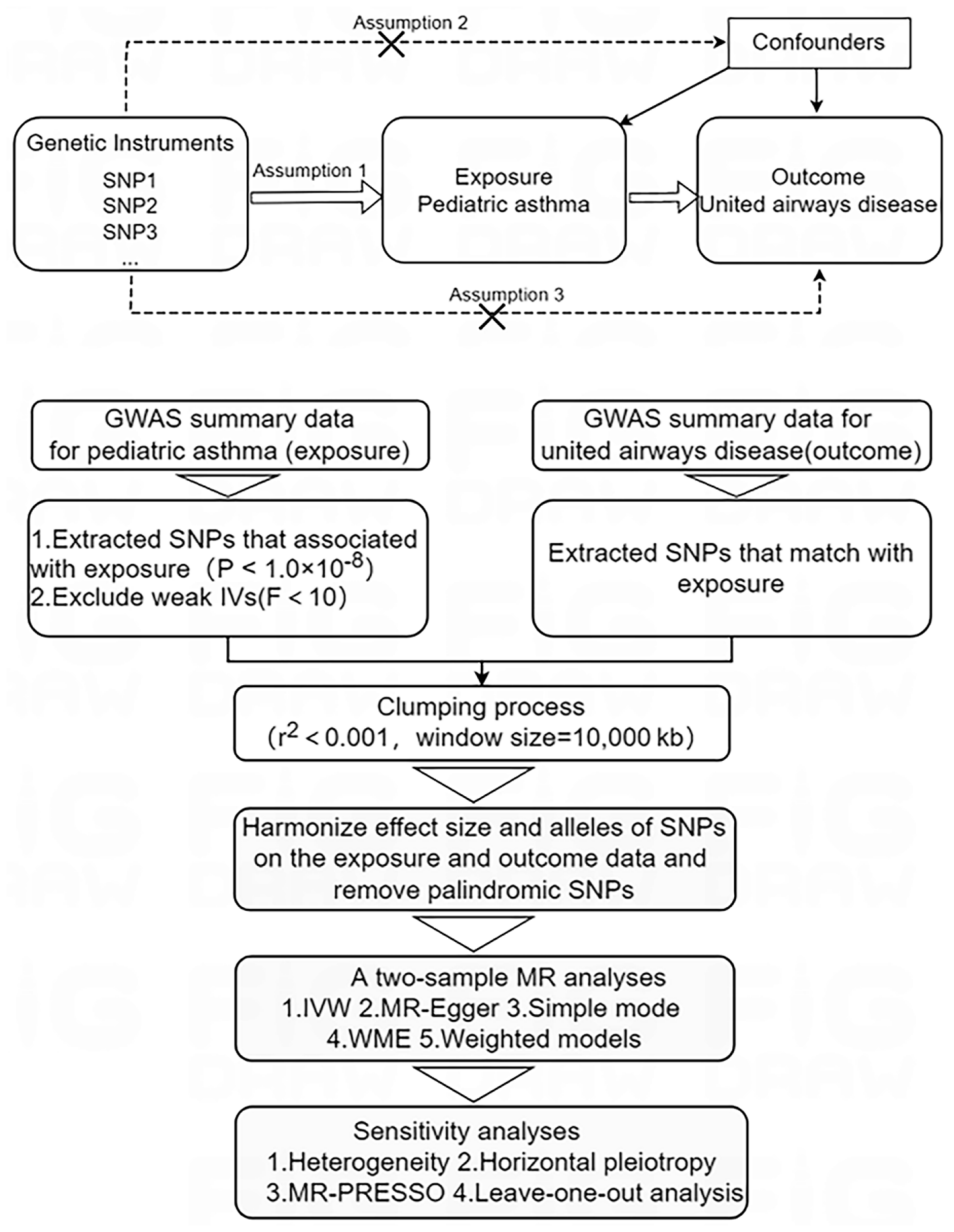
Study design overview. IVs, instrumental variables; MR, Mendelian randomization; SNPs, single nucleotide polymorphisms. Mendelian randomization study design and assumptions. Assumption 1: relevance assumption; Assumption 2: independence assumption; Assumption 3: exclusivity assumption.

The two-sample MR approach was employed to investigate the causal impact of pediatric asthma on UAD. MR analysis primarily employs the inverse variance weighting (IVW) method to infer causal effects between exposure and outcome. In this study, pediatric asthma was used as the exposure factor, single nucleotide polymorphisms (SNPs) significantly associated with pediatric asthma were used as instrumental variables (IVs), and UAD was considered as the outcome factor. MR analysis employs genetic variants to assess causality of observational data and IVs included in the study must satisfy three key assumptions: firstly, relevance assumption: there is a robust and strong association with the exposure factor; secondly, independence assumption: there is no confounding variable between exposure and outcome; thirdly, exclusivity assumption: it should not have any direct relationship with the outcome, but only affect it through exposure (21, 22). The study design overview is presented in Figure 1.

### 2.2 Data sources

The genome-wide association studies (GWAS) summary data for pediatric asthma was selected as the exposure factor, while the GWAS summary data for seven common UAD were chosen as outcome factors, including chronic sinusitis, chronic rhinitis, nasopharyngitis and pharyngitis, chronic diseases of tonsils and adenoids, chronic laryngitis and laryngotracheitis, chronic bronchitis, bronchiectasis, and COPD (Table 1 and Supplementary material). The GWAS data utilized in this study were exclusively sourced from the Finn Gen and European Bioinformatics Institute (EBI) databases. The primary goal of the Finn Gen database is to collect and analyze genomic data from 500,000 Finns, integrating it with national health registry data to reveal relationships between genetic variations and diseases, thus advancing genetics in human health (23). The EBI database encompasses an extensive array of genotype and phenotype data, providing a multitude of data analysis and integration tools, thereby advancing applications and developments in genomics, proteomics, transcriptomics, and other related fields (24). The GWAS data on pediatric asthma was obtained from the EBI database, comprising 27,712 cases and 411,131 controls. The GWAS data on chronic sinusitis was obtained from the EBI database, comprising 3,014 cases and 481,584 controls. The GWAS data on chronic rhinitis, nasopharyngitis and pharyngitis was obtained from the Finn Gen database, comprising 5,355 cases and 167,849 controls. The GWAS data on chronic diseases of tonsils and adenoids was obtained from the Finn Gen database, comprising 24,463 cases and 167,849 controls. The GWAS data on chronic laryngitis and laryngotracheitis was obtained from the Finn Gen database, comprising 2,138 cases and 167,849 controls. The GWAS data on chronic bronchitis was obtained from the EBI database, comprising 10,159 cases and 440,263 controls. The GWAS data on bronchiectasis was obtained from the EBI database, comprising 2,888 cases and 440,263 controls. The summary dataset on COPD was obtained from the EBI database, comprising 13,530 cases and 454,945 controls. To mitigate potential bias arising from confounding factors within the population, we specifically limited our study to individuals of European ancestry. The pooled data utilized in this investigation were obtained from publicly available summary data of GWAS, thereby obviating the need for additional ethical approval.

**Table 1:** Overview of study data.

Trait	Data type	Consortium	GWAS ID	Case	Control	SNPs	Ethnicity	Year
Pediatric asthma	Exposure	EBI database	ebi-a-GCST90018895	27,712	411,131	24,166,696	European	2021
Chronic sinusitis	Outcome	EBI database	ebi-a-GCST90038673	3,014	481,584	9,587,836	European	2021
Chronic rhinitis, nasopharyngitis and pharyngitis	Outcome	Finn Gen database	finn-b-J10_CHRONRHINITIS	5,355	167,849	16,380,284	European	2021
Chronic diseases of tonsils and adenoids	Outcome	Finn Gen database	finn-b-J10_CHRONTONSADEN	24,463	167,849	16,380,381	European	2021
Chronic laryngitis and laryngotracheitis	Outcome	Finn Gen database	finn-b-J10_CHRONLARYNGITIS	2,138	167,849	16,380,258	European	2021
Chronic bronchitis	Outcome	EBI database	ebi-a-GCST90018824	10,159	440,263	24,182,745	European	2021
Bronchiectasis	Outcome	EBI database	ebi-a-GCST90018801	2,888	440,263	24,189,609	European	2021
COPD	Outcome	EBI database	ebi-a-GCST90018807	13,530	454,945	24,180,654	European	2021

### 2.3 IV selection

IVs necessitate three fundamental assumptions that are in accordance with the principles of MR. Firstly, SNPs associated with pediatric asthma, meeting the locus-wide significance threshold (*p*-value <1.0 × 10^−8^), were identified as potential IVs; secondly, to evaluate the robustness of IVs and minimize potential bias from weak IVs, we calculated the *F*-statistic for each SNPs, excluding SNPs with an *F*-value less than 10 (25); thirdly, we performed Linkage disequilibrium-based clumping procedure (*r*^2^ < 0.001 and window size = 10,000 kb) to ensure the independence of each IVs. Linkage disequilibrium estimation was conducted utilizing the 1,000 Genomes European reference panel (26); fourthly, harmonize the exposure and outcome data, ensuring that the effect size for exposure and outcome correspond to the same effect allele, while removing palindrome SNPs with intermediate allele frequencies (27). The study flow overview is depicted in Figure 1.

### 2.4 Statistical analysis

The MR approach was employed in this study to investigate the causal relationship between pediatric asthma and seven common UAD. Five two-sample MR analysis methods, namely inverse variance weighted (IVW), MR-Egger regression, Simple mode, weighted median (WME), and weighted model, were utilized with a significance level of *p*-value <0.05 indicating statistical significance. The primary inference drawn from the MR analysis is predominantly based on the IVW outcomes, while other analytical approaches are employed as supplementary evidence to ensure result robustness (28). IVW can integrate the Wald estimates of individual SNPs to derive a comprehensive estimate, thereby facilitating robust and accurate causal assessment (29, 30). The association between pediatric asthma and UAD risk is quantified using odds ratio along with its 95% confidence interval (CI), where a significance level of *p*-value <0.05 indicates a causal relationship. Additionally, scatter plots and forest plots are employed to visually present the findings of the MR analysis. The statistical analysis in this study was conducted using R (version 4.3.1) and the Two-Sample MR package (version 0.5.7), as well as the MRPRESSO package (version 1.0).

### 2.5 Sensitivity analysis

Sensitivity analysis mainly includes tests for heterogeneity, tests for horizontal pleiotropy, MR-PRESSO test, and leave-one-out test. Heterogeneity testing will be conducted using IVW and MR-Egger regression methods, while the Cochran *Q* statistic will be calculated to evaluate the extent of heterogeneity. If the *p*-value is less than 0.05, it indicates heterogeneity, leading to subsequent analysis employing a random effects model. Conversely, if the *p*-value is greater than or equal to 0.05, a fixed effects model will be utilized (31). Using MR-Egger regression for testing horizontal pleiotropy, if the *p*-value is less than 0.05, it suggests the presence of horizontal pleiotropy. The MR-PRESSO method is employed to detect the presence of outliers, and if any are identified, the causal estimates undergo reassessment subsequent to their removal. The leave-one-out analysis is a systematic method employed to ascertain whether the MR result is influenced by an individual SNPs, through the deliberate exclusion of each SNPs in a sequential manner (32).

## 3 Results

### 3.1 Results of IV screening

**Figure 2:**
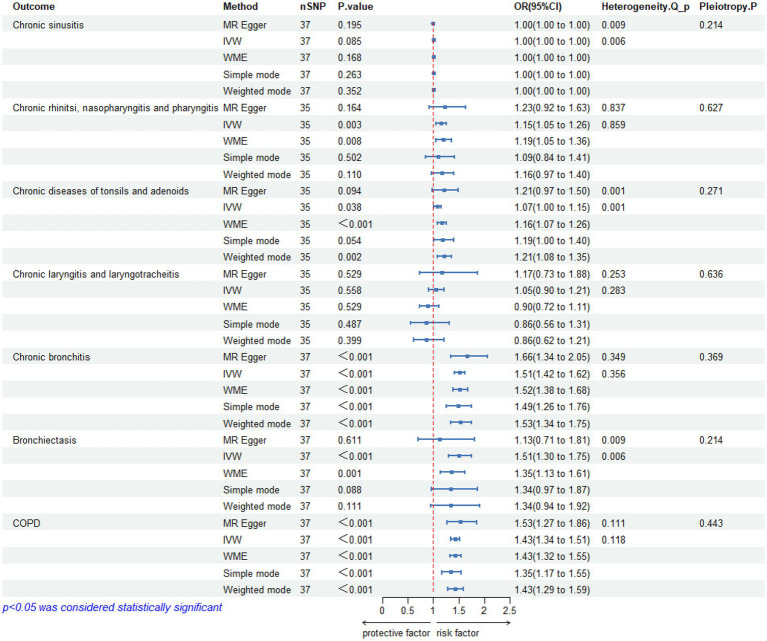
Results of Mendelian randomization studies, heterogeneity analysis and pleiotropy between pediatric asthma and UAD. CI, confidence interval; OR, odds ratio; SNP, single nucleotide polymorphism; IVW, inverse variance weighted; WME, weighted median; *Q*, Cochran’s *Q* statistic; COPD, chronic obstructive pulmonary disease.

According to the pre-established criteria, SNPs that simultaneously satisfied all three major hypotheses were selected after undergoing a series of rigorous quality control steps. The *F*-values of the SNPs included in the analysis ranged from 30.43 to 287.58, and no evidence of weak instrumental variable bias was observed (Supplementary Table S1). Detailed information regarding the final number of included SNPs is provided in Figure 2.

### 3.2 Results of MR analysis

#### 3.2.1 Pediatric asthma and chronic sinusitis

The IVW analysis did not reveal a significant causal relationship between pediatric asthma and chronic sinusitis (OR = 1.00, 95%CI:1.00–1.00, *p*-value = 0.085), and was replicated via MR-Egger (OR = 1.00, 95%CI: 1.00–1.00, *p*-value = 0.195), WME (OR = 1.00, 95%CI: 1.00–1.00, *p*-value = 0.168), Simple mode (OR = 1.00, 95%CI: 1.00–1.00, *p*-value = 0.263), weighted mode (OR = 1.00, 95%CI: 1.00–1.00, *p*-value = 0.352).

**Figure 3:**
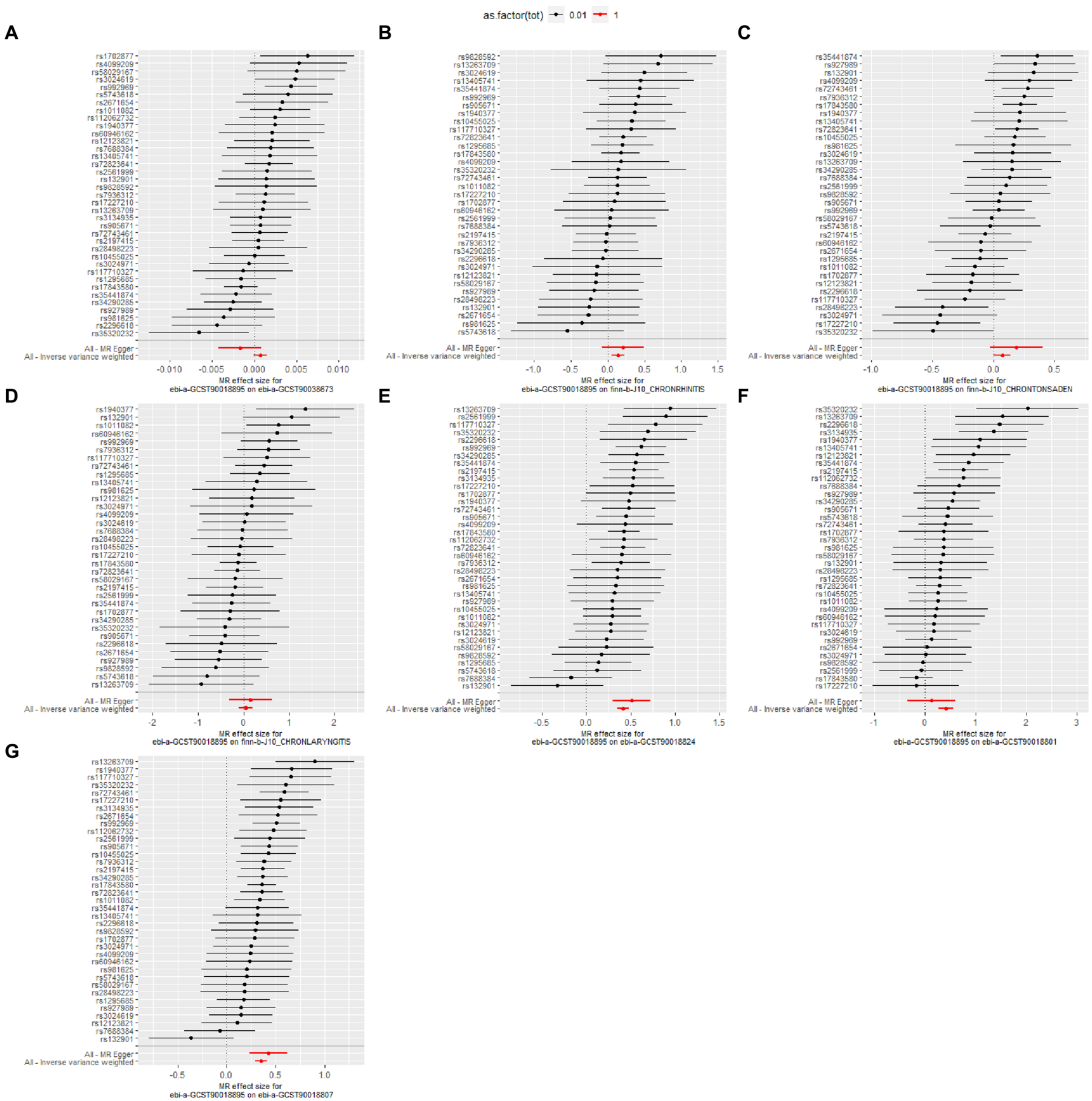
Leave-one-out analysis for the causal effect of pediatric asthma on the risk of UAD. **(A)** Chronic sinusitis; **(B)** Chronic rhinitis, nasopharyngitis and pharyngitis; **(C)** Chronic diseases of tonsils and adenoids; **(D)** Chronic laryngitis and laryngotracheitis; **(E)** Chronic bronchitis; **(F)** Bronchiectasis; **(G)** Chronic obstructive pulmonary disease.

The heterogeneity test results revealed that both MR-Egger (*p*-value = 0.009) and IVW (*p*-value = 0.006) exhibited *p*-value <0.05, indicating the presence of heterogeneity in the data set, so the random-effect model was employed for analysis. The horizontal pleiotropy test revealed no evidence of horizontal pleiotropy (*p*-value = 0.214 > 0.05). No outliers were detected by MR-PRESSO test. Leave-one-out analyses did not identify any SNPs exerting a substantial impact on the estimated causal associations (Figures 2, 3).

#### 3.2.2 Pediatric asthma and chronic rhinitis, nasopharyngitis, and pharyngitis

The IVW analysis found a significant causal association between pediatric asthma and chronic rhinitis, nasopharyngitis and pharyngitis (OR = 1.15, 95%CI (1.05–1.26), *p*-value = 0.003), and was replicated via WME (OR = 1.19, 95%CI (1.05–1.36), *p*-value = 0.008), but MR-Egger (OR = 1.23, 95%CI: 0.92–1.63, *p*-value = 0.164), Simple mode (OR = 1.09, 95%CI: 0.84–1.41, *p*-value = 0.502) and weighted mode (OR = 1.16, 95%CI: 0.97–1.40, *p*-value = 0.110) did not support the IVW results.

The heterogeneity test results revealed that both MR-Egger (*p*-value = 0.837) and IVW (*p*-value = 0.859) exhibited *p*-value >0.05, indicating the absence of heterogeneity in the data set, so the fixed-effect model was employed for analysis. The horizontal pleiotropy test revealed no evidence of horizontal pleiotropy (*p*-value = 0.627 > 0.05). No outliers were detected by MR-PRESSO test. Leave-one-out analyses did not identify any SNPs exerting a substantial impact on the estimated causal associations (Figures 2, 3).

#### 3.2.3 Pediatric asthma and chronic diseases of tonsils and adenoids

A significant causal relationship between pediatric asthma and chronic diseases of tonsils and adenoids was observed in IVW analysis (OR = 1.07, 95%CI: 1.00–1.15, *p*-value = 0.038), which could be confirmed by WME (OR = 1.16, 95%CI: 1.07–1.26, *p*-value <0.001), and weighted mode (OR = 1.21, 95%CI: 1.08–1.35, *p*-value = 0.002), but MR-Egger (OR = 1.21, 95%CI: 0.97–1.50, *p*-value = 0.094), and Simple mode (OR = 1.19, 95%CI: 1.00–1.40, *p*-value = 0.054) did not support the IVW results.

The heterogeneity test results revealed that both MR-Egger (*p*-value = 0.001) and IVW (*p*-value = 0.001) exhibited *p*-value <0.05, indicating the presence of heterogeneity in the data set, so the random-effect model was employed for analysis. The horizontal pleiotropy test revealed no evidence of horizontal pleiotropy (*p*-value = 0.271 > 0.05). No outliers were detected by MR-PRESSO test. Leave-one-out analyses did not identify any SNPs exerting a substantial impact on the estimated causal associations (Figures 2, 3).

#### 3.2.4 Pediatric asthma and chronic laryngitis and laryngotracheitis

The IVW analysis did not reveal any significant causal relationship between pediatric asthma and chronic laryngitis and laryngotracheitis (OR = 1.05, 95%CI: 0.90–1.21, *p*-value = 0.558). This finding is consistent with the conclusions drawn from MR-Egger (OR = 1.17, 95%CI: 0.73–1.88, *p*-value = 0.529), WME (OR = 0.09, 95%CI: 0.72–1.11, *p*-value = 0.529), Simple mode (OR = 0.86, 95%CI: 0.56–1.31, *p*-value = 0.487), and weighted mode (OR = 0.86, 95%CI: 0.62–1.21, *p*-value = 0.399).

The heterogeneity test results revealed that both MR-Egger (*p*-value = 0.253) and IVW (*p*-value = 0.283) exhibited *p*-value >0.05, indicating the absence of heterogeneity in the data set, so the random-effect model was employed for analysis. The horizontal pleiotropy test revealed no evidence of horizontal pleiotropy (*p*-value = 0.636 > 0.05). No outliers were detected by MR-PRESSO test. Leave-one-out analyses did not identify any SNPs exerting a substantial impact on the estimated causal associations (Figures 2, 3).

#### 3.2.5 Pediatric asthma and chronic bronchitis

The IVW analysis revealed a significant causal association between pediatric asthma and chronic bronchitis (OR = 1.51, 95% CI: 1.42–1.62, *p*-value <0.001). This finding is corroborated by other methods including MR-Egger (OR = 1.66, 95% CI: 1.34–2.05, *p* < 0.001), WME (OR = 1.52, 95%CI: 1.38–1.68, *p*-value < 0.001), Simple mode (OR = 1.49, 95%CI: 1.26–1.76, *p*-value < 0.001), and weighted mode (OR = 1.53, 95 %CI: 1.34–1.75, *p*-value < 0.00 L).

The heterogeneity test results revealed that both MR-Egger (*p*-value = 0.349) and IVW (*p*-value = 0.356) exhibited *p*-value >0.05, indicating the absence of heterogeneity in the data set, so the fixed-effect model was employed for analysis. The horizontal pleiotropy test revealed no evidence of horizontal pleiotropy (*p*-value = 0.369 > 0.05). No outliers were detected by MR-PRESSO test. Leave-one-out analyses did not identify any SNPs exerting a substantial impact on the estimated causal associations (Figure 2).

#### 3.2.6 Pediatric asthma and bronchiectasis

A significant causal relationship between pediatric asthma and bronchiectasis was observed in IVW analysis (OR = 1.51, 95%CI: 1.30–1.75, *p*-value <0.001), which could be confirmed by WME (OR = 1.35, 95%CI: 1.13–1.61, *p*-value = 0.001). But MR-Egger (OR = 1.13, 95%CI: 0.71–1.81, *p*-value = 0.611), Simple mode (OR = 1.34, 95%CI: 0.97–1.87, *p*-value = 0.088), weighted mode (OR = 1.34, 95%CI: 0.94–1.92, *p*-value = 0.111) did not support the IVW results.

The heterogeneity test results revealed that both MR-Egger (*p*-value = 0.009) and IVW (*p*-value = 0.006) exhibited *p*-value <0.05, indicating the presence of heterogeneity in the data set, so the random-effect model was employed for analysis. The horizontal pleiotropy test revealed no evidence of horizontal pleiotropy (*p*-value = 0.214 > 0.05). MR-PRESSO test detected one outlier (rs17843580). Leave-one-out analyses did not identify any SNPs exerting a substantial impact on the estimated causal associations (Figures 2, 3).

When the MR analysis was repeated after the outliers were removed, the significant causal relationship between pediatric asthma and bronchiectasis was still observed in the IVW analysis (OR = 1.63, 95%CI: 1.42–1.88, *p*-value <0.001), which could be confirmed by the WME (OR = 1.45, 95%CI: 1.21–1.74, *p*-value <0.001) were replicated, but MR-Egger (OR = 1.69, 95%CI: 1.01–2.83, *p*-value = 0.052), Simple mode (OR = 1.35, 95%CI: 0.97–1.86, *p*-value = 0.081), weighted model (OR = 1.35, 95%CI: 1.00–1.81, *p*-value = 0.056) did not support the IVW results (Supplementary material).

#### 3.2.7 Pediatric asthma and COPD

The IVW analysis reported a significant causal relationship between pediatric asthma and COPD (OR = 1.43, 95%CI: 1.34–1.51, *p*-value <0.001), and was consistent with the findings of the MR-Egger (OR = 1.53, 95%CI: 1.27–1.86, *p*-value <0.001), WME (OR = 1.43, 95%CI: 1.32–1.55, *p*-value <0.001), Simple mode (OR = 1.35, 95%CI: 1.17–1.55, *p*-value <0.001), and weighted mode (OR = 1.43, 95%CI: 1.29–1.59, *p*-value <0.001).

The heterogeneity test results revealed that both MR-Egger (*p*-value = 0.111) and IVW (*p*-value = 0.118) exhibited *p*-value >0.05, indicating the absence of heterogeneity in the data set, so the fixed-effect model was employed for analysis. The horizontal pleiotropy test revealed no evidence of horizontal pleiotropy (*p*-value = 0.443 > 0.05). No outliers were detected by MR-PRESSO test. Leave-one-out analyses did not identify any SNPs exerting a substantial impact on the estimated causal associations (Figures 2, 3).

**Figure 4:**
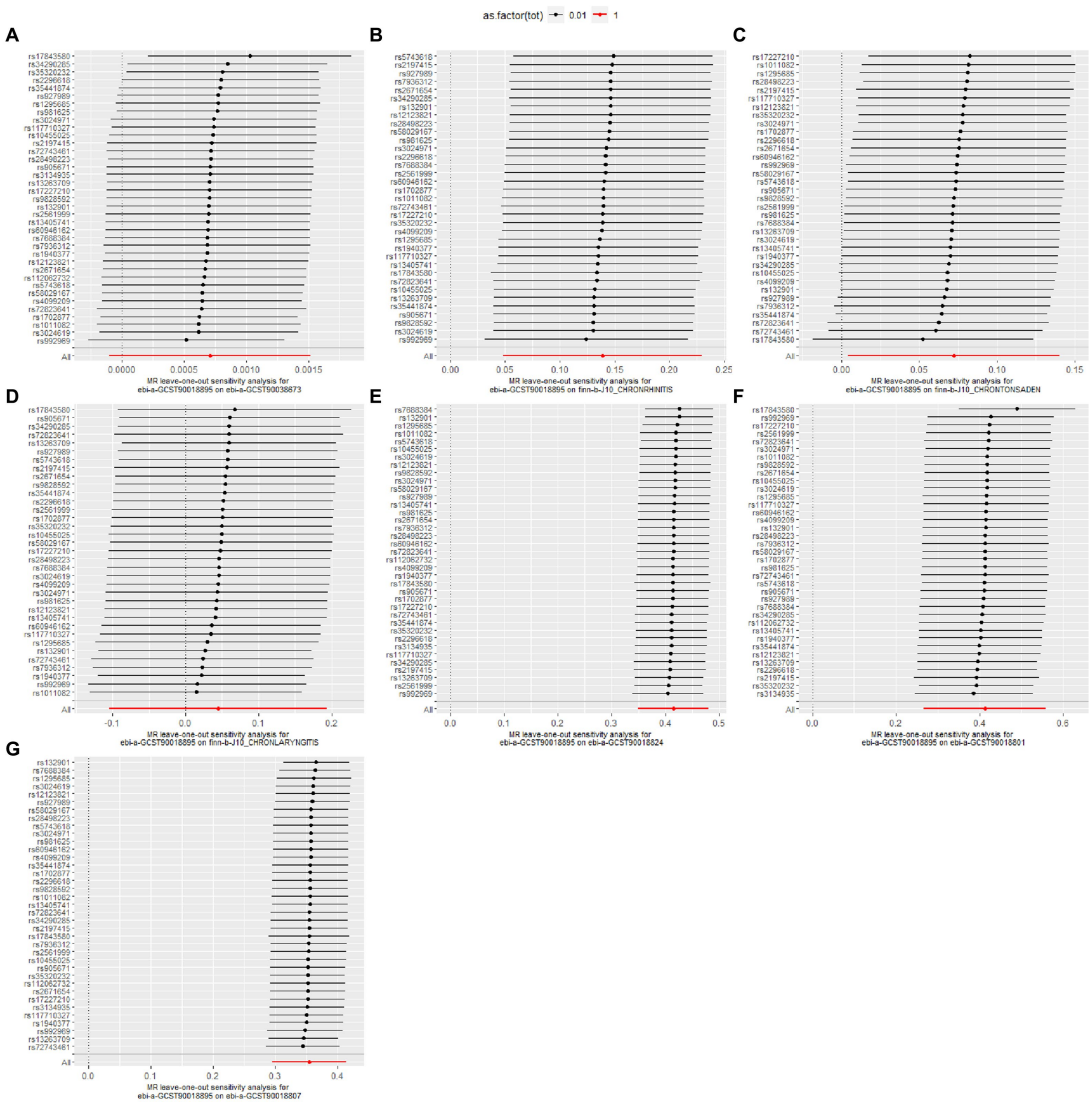
Forest plot for the causal effect of pediatric asthma on the risk of UAD. **(A)** Chronic sinusitis; **(B)** Chronic rhinitis, nasopharyngitis and pharyngitis; **(C)** Chronic diseases of tonsils and adenoids; **(D)** Chronic laryngitis and laryngotracheitis; **(E)** Chronic bronchitis; **(F)** Bronchiectasis; **(G)** Chronic obstructive pulmonary disease.

**Figure 5:**
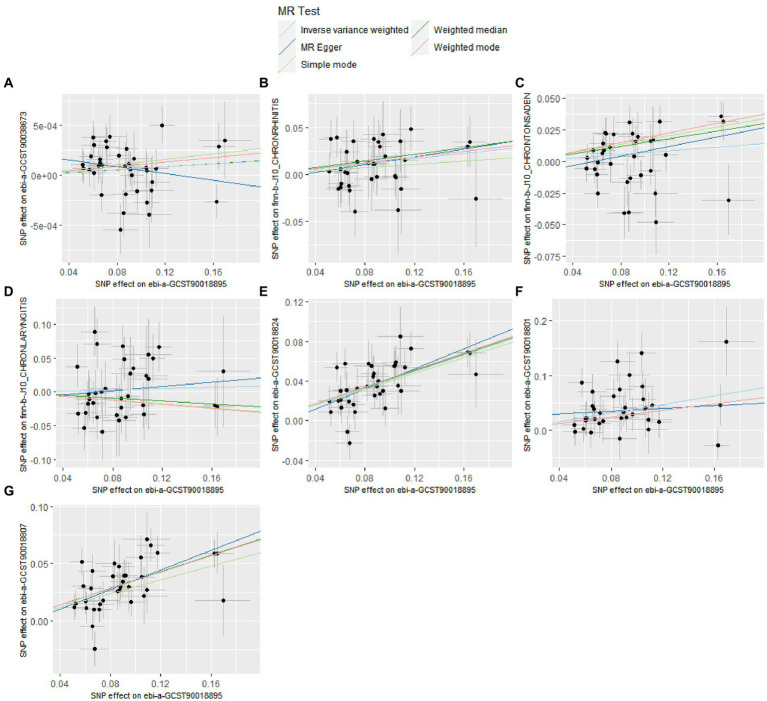
Scatter plot for the causal effect of pediatric asthma on the risk of UAD. **(A)** Chronic sinusitis; **(B)** Chronic rhinitis, nasopharyngitis and pharyngitis; **(C)** Chronic diseases of tonsils and adenoids; **(D)** Chronic laryngitis and laryngotracheitis; **(E)** Chronic bronchitis; **(F)** Bronchiectasis; **(G)** Chronic obstructive pulmonary disease.

The details of leave-one-out analysis are shown in Figure 3. The forest plot and scatter plot illustrating the causal relationships between genetically predicted pediatric asthma and the risk of UAD are presented in Figures 4, 5. The detailed estimates of causal effects.

## 4 Discussion

To the best of our knowledge, this study represents the first investigation in the field of MR research to examine the causal impact of pediatric asthma on UAD. Our findings demonstrated a significant causal relationship between pediatric asthma and the occurrence of chronic rhinitis, nasopharyngitis, pharyngitis, chronic diseases of tonsils and adenoids, chronic bronchitis, bronchiectasis, as well as COPD. However, there was no discernible causal relationship observed between pediatric asthma and the occurrence of chronic sinusitis, chronic laryngitis, or laryngitis.

The concept of UAD was initially proposed in the late 1990s, positing that the respiratory tract constitutes a cohesive entity with numerous shared anatomical and histological characteristics, and the lesions in any region of the airway will exert an impact on the function of the whole airway (33). The concept is grounded in the shared pathophysiological and immunological mechanisms that underlie specific respiratory diseases (34). Persistent and increased inflammation is a common pathological characteristic of UAD, accompanied by significant genetic modifications and stringent gene regulation (35). In the context of allergic inflammation, upper and lower airway diseases exhibit overlapping immunopathological features, including activation of epithelial immune responses, infiltration of eosinophils, production of Ig E antibodies, and activation of mast cells (36). Studies have shown that nasal allergen provocation in allergic rhinitis not only increases nasal eosinophilia but also induces bronchial eosinophilic inflammation, bronchoconstriction, and bronchial hyperresponsiveness, indicating inflammatory crosstalk between the upper and lower airways (37).

The primary pathological mechanisms of pediatric asthma include chronic airway inflammation, airway hyperresponsiveness, and airway remodeling. These pathological processes undoubtedly affect airway function to varying degrees and may be potential contributing factors for the development of UAD in later life. Airway Inflammation: Airway inflammatory responses are endogenous factors in the progression of pediatric asthma and UAD, involving the infiltration of inflammatory cells and the release of inflammatory mediators. These processes are dynamic and can be influenced by treatment, infection, environmental exposure, and disease progression characteristics (8). For instance, eosinophilic airway inflammation associated with TH2 cytokines (IL-4, IL-5, and IL-13) and/or Ig E is an underlying pathological mechanism in both chronic rhinosinusitis with nasal polyps and asthma (38). Inflammation observed in Asthma-COPD Overlap Syndrome (ACOS) is primarily driven by eosinophils and neutrophils, as evidenced by studies demonstrating significantly elevated levels of IL-6 in ACOS patients compared to healthy individuals and those with asthma (39). Airway Remodeling: Prolonged chronic airway inflammation can lead to mucosal swelling and excessive mucus secretion, resulting in structural changes and remodeling of the airways. This phenomenon can be observed in both pediatric asthma and UAD. Airway remodeling phenomena, such as thickening of the airway wall, damage to epithelial cells, hyperplasia of goblet cells, and proliferation of smooth muscle cells, are observed in both asthma and COPD (40, 41). In severe corticosteroid-dependent asthma, there can be an increase in the number and area occupied by bronchial blood vessels—a condition that is also seen in severe COPD and bronchiectasis (40). Airway Hyperresponsiveness: Airway hyperresponsiveness refers to an exaggerated sensitivity of the airways to stimuli, such as allergens or irritants, resulting in bronchospasm and constriction. It is a crucial clinical feature in pulmonary diseases (allergic rhinitis, asthma, COPD) and is closely related to airway inflammation, remodeling, and mucus secretion, playing a significant role in the progression of UAD (42, 43).

Pediatric asthma exhibits a strong correlation with upper respiratory conditions, including chronic rhinitis, chronic diseases of the tonsils and adenoids. Firstly, both of these conditions are frequently observed as comorbidities in asthma, with chronic rhinitis being diagnosed in more than a quarter of children with asthma (44). Secondly, chronic inflammation plays a pivotal role of airway remodeling and irreversible airflow limitation in patients with long-term asthma, and in the upper respiratory tract, it can not only cause chronic rhinitis, but also stimulate adenoids and tonsils, leading to hyperplasia and hypertrophy (45, 46). Furthermore, the occurrence and progression of these diseases are intricately linked to prolonged exposure to nasal allergens (47). Similarly, pediatric asthma exhibits a robust association with lower respiratory conditions, including chronic bronchitis, bronchiectasis, and COPD. Although chronic bronchitis primarily occurs in middle-aged and elderly individuals, it may have its origins in childhood. Research indicates that childhood asthma can serve as a clinical predictive factor for adult chronic bronchitis (48). Moreover, recent data indicates a tenfold increase in the risk of developing chronic bronchitis among current asthma patients compared to two decades ago (49). A mate analysis revealed that the average prevalence of bronchiectasis among patients with asthma was determined to be 36.6% (50). Bronchiectasis is a rare occurrence in the pediatric population and is typically considered as the ultimate result of many years of chronic, poorly controlled asthma. However, findings from a cross-sectional study indicate that approximately one-third of children with severe asthma exhibit bronchiectasis (51). Furthermore, research indicates that eosinophilic airway inflammation plays a crucial role in the formation and development of bronchiectasis in asthmatic patients, characterized by elevated peripheral blood eosinophil count and sputum eosinophil percentage (52). The findings of a 50 year cohort study demonstrate a significant association between pediatric asthma and an increased risk of developing COPD in later life (53); children with a prior history of asthma exhibit a 3.45-fold increased likelihood of developing COPD compared to children without such a medical background (54). Pediatric asthma can impair lung development, hindering the attainment of normal peak lung function during adolescence and diminishing adult lung function, thereby augmenting the susceptibility to developing COPD (55). Nevertheless, early suppression of airway inflammation and prevention of asthma exacerbations in childhood can foster optimal lung development and maturation, culminating in enhanced lung function and a reduced risk of COPD in adulthood (55). In a cross-sectional survey conducted on 851 patients with chronic sinusitis in seven cities across China, the prevalence of Chronic sinusitis among individuals with asthma was observed to be 24%, which is significantly higher compared to the 7% prevalence found among non-asthmatic participants (56). However, our research findings do not provide evidence for a causal relationship between childhood asthma and chronic sinusitis. Furthermore, further investigation is warranted to determine whether there exists a reverse causal link between these two conditions (57). Chronic laryngitis and laryngotracheitis, although categorized as respiratory disorders, are commonly linked to factors such as pharyngolaryngeal reflux, prolonged exposure to irritants, excessive vocal cord use, and bacterial or viral infections. There are significant differences in the pathogenesis compared to pediatric asthma. Likewise, our research results do not lend support to the existence of a causal relationship between the two diseases (58).

Pediatric asthma and UAD are closely linked in terms of disease risk factors, epidemiology, and pathological mechanisms. Conventional observational studies encounter challenges in effectively mitigating exposure measurement errors, confounding bias, and reverse causality, necessitating substantial time and social resources. MR analysis is an efficient causal analysis method that effectively mitigates potential individual differences and biases in intervention allocation by randomly assigning study participants to different treatment groups, thereby ensuring the reliability and generalizability of research findings (59). This study contributes to enhance our understanding of UAD and facilitates accurate predictions regarding the developmental trends and long-term outcomes of asthma in children. For instance, our study establishes a causal association between pediatric asthma and COPD. A large cohort study indicates that even clinically remitted childhood asthma may remain a significant risk factor for late-life COPD, consistent with our conclusions (16). The 2023 Global Initiative for Asthma statement emphasizes the importance of managing and identifying asthma comorbidities. Developing specific treatment strategies based on the UAD concept to manage asthma and its comorbidities could enhance treatment efficacy, reduce medication use, and save medical resources (1). Biologics such as dupilumab, omalizumab, and mepolizumab have been approved for severe asthma and severe chronic rhinosinusitis with nasal polyps. These medications improve asthma symptoms and lung function, reduce disease recurrence, and streamline medication regimens, demonstrating broad potential in the treatment of UAD (34).

Leveraging the unique advantages of the MR method, our study systematically assessed the causal relationship between pediatric asthma and UAD. This approach emulates randomized controlled trials within an observational study framework and incorporates multiple sensitivity analyses, thereby augmenting the scientific validity and robustness of our findings (30). This study partially validated previous observational research, deepening our understanding of the “one airway, one disease” concept. However, there remains a paucity of scientific evidence on the integrated management of pediatric asthma and UAD. Most clinical trials and real-world studies regard asthma or UAD as independent conditions or comorbidities, rather than exploring these diseases based on the concept of UAD. Further experimental and observational studies are needed to elucidate the pathological mechanisms underlying these causal associations, to promote the advancement of preventive and therapeutic systems for asthma and UAD. Furthermore, certain limitations of this MR study should be acknowledged: Firstly, due to the unavailability of raw GWAS data, more specific phenotypes such as gender, age and asthma severity could not be explored, and further subgroup analysis could not be carried out, which may lead to bias of the study results; Secondly, the data utilized in this study exclusively originated from European populations, thus the generalizability of the findings to other populations may be constrained; Thirdly, MR is a robust approach for investigating causal relationships without the need to examine underlying biological mechanisms, and its research findings necessitate further rigorous validation.

## 5 Conclusion

In conclusion, our findings suggest that pediatric asthma may be a potential risk factor for various UAD, underscoring the significance of the “one airway, one disease” concept in the prevention and management of chronic respiratory diseases. Additionally, elucidating the mechanisms underlying the causal relationship between pediatric asthma and UAD is crucial for the comorbidity management of pediatric asthma and the diagnosis and prevention of UAD.

## Data availability statement

Publicly available datasets were analyzed in this study. This data can be found here: relevant tables are included in the manuscript.

## Ethics statement

Ethical approval was not required for the study involving humans in accordance with the local legislation and institutional requirements. Written informed consent to participate in this study was not required from the participants or the participants’ legal guardians/next of kin in accordance with the national legislation and the institutional requirements.

## Author contributions

TG: Software, Writing – original draft, Formal analysis, Methodology. QC: Writing – original draft, Investigation, Methodology. SH: Supervision, Validation, Writing – review & editing. RZ: Writing – original draft, Formal analysis. JW: Writing – original draft, Formal analysis.
