# Supplementary material for: Causal associations between pediatric asthma and united airways disease: a two-sample Mendelian randomization analysis
Source: Front Med (Lausanne). 2024 Jun 10;11:1369695. doi: 10.3389/fmed.2024.1369695 (PMC11196945; doi:10.3389/fmed.2024.1369695)
Supplement: Supplementary file 1 [file Data_Sheet_1.docx]

Supplementary Material

Supporting information for

**Causal associations between pediatric asthma and united airways disease: A two-sample Mendelian randomization analysis**

# Supplementary Table

**Supplementary Table 1.** Supplementary information on the study data

| **Trait** | **GWAS ID** | **Registry filters** | **Data sources** | **PMID** |
| --- | --- | --- | --- | --- |
| Pediatric asthma | ebi-a-GCST90018895 | ICD-10 | [https://gwas.mrcieu.ac.uk/](https://gwas.mrcieu.ac.uk/) | 34594039 |
| Chronic sinusitis | ebi-a-GCST90038673 | ICD-10, ICD-9 |  | 33959723 |
| Chronic bronchitis | ebi-a-GCST90018824 | ICD-10 |  | 34594039 |
| Bronchiectasis | ebi-a-GCST90018801 | ICD-10 |  | 34594039 |
| COPD | ebi-a-GCST90018807 | ICD-10 |  | 34594039 |
| Chronic rhinitis, nasopharyngitis and pharyngitis | finn-b-J10_CHRONRHINITIS | ICD-10.J31, ICD-9.472, ICD-8.502 | <https://www.finngen.fi/en> |  |
| Chronic diseases of tonsils and adenoids | finn-b-J10_CHRONTONSADEN | ICD-10.J35, ICD-9.474, ICD-8.500 |  |  |
| Chronic laryngitis and laryngotracheitis | finn-b-J10_CHRONLARYNGITIS | ICD-10.J37, ICD-9.476, ICD-8.506 |  |  |

## **Supplementary Figure**

**Supplementary Figure 1.** Funnel plot for the causal effect of pediatric asthma on the risk of UAD.





FIGURE 1.

Funnel plot for the causal effect of pediatric asthma on the risk of UAD. (A) Chronic sinusitis; (B) Chronic rhinitsi, nasopharyngitis and pharyngitis; (C) Chronic diseases of tonsils and adenoids; (D) Chronic laryngitis and laryngotracheitis; (E) Chronic bronchitis; (F) Bronchiectasis; (G) Chronic obstructive pulmonary disease.

# Supplementary Data

MR-PRESSO test of pediatric asthma and bronchiectasis detected one outlier (rs17843580). After excluding outliers, we conducted MR analysis and sensitivity analysis again. The obtained results are presented below.

**Supplementary Figure 2.** The results of mendelian randomization studies, heterogeneity analysis and pleiotropy.

**
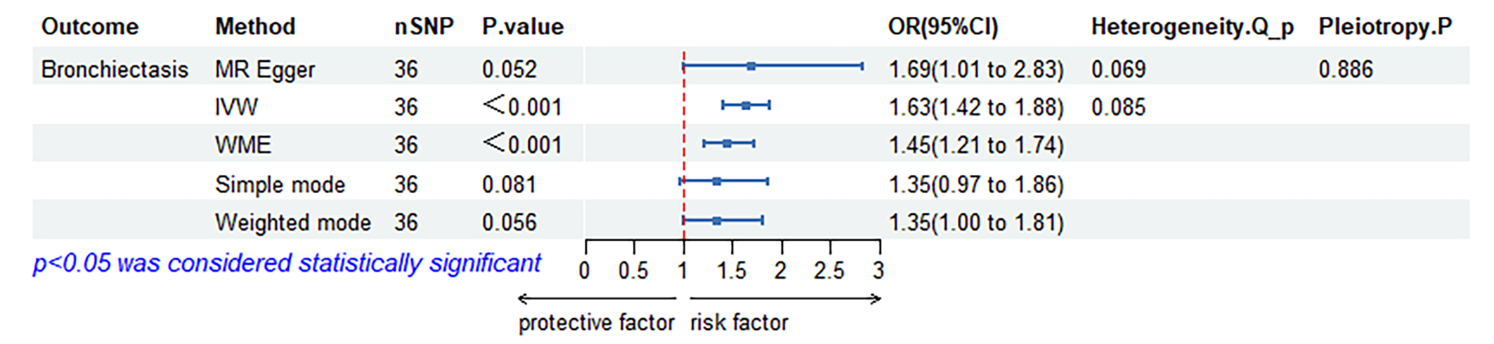
**

FIGURE 2

Results of mendelian randomization studies, heterogeneity analysis and pleiotropy between pediatric asthma and bronchiectasis. CI =confidence interval, OR=odds ratio, SNP=single nucleotide polymorphism, IVW=inverse variance weighted, WME=weighted median, Q=Cochran’s Q statistic.

**Supplementary Figure 3.** Leave-one-out analysis plot.





FIGURE 3.

Leave-one-out analysis for the causal effect of pediatric asthma on the risk of bronchiectasis.

**Supplementary Figure 4.** Forest plot.





FIGURE 4.

Forest plot for the causal effect of pediatric asthma on the risk of bronchiectasis.

**Supplementary Figure 5.** Scatter plot.





FIGURE 5.

Scatter plot for the causal effect of pediatric asthma on the risk of bronchiectasis.

**Supplementary Figure 6.** Funnel plot.

**
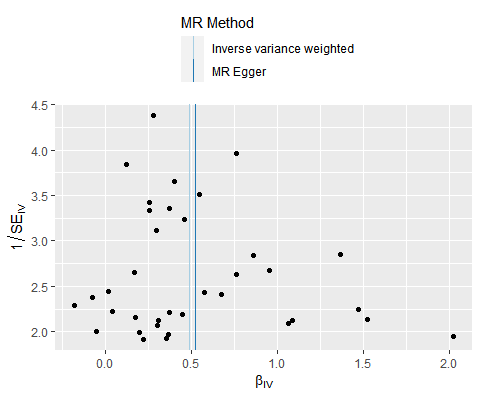
**

FIGURE 6.

Funnel plot for the causal effect of pediatric asthma on the risk of bronchiectasis.
